# Comparison effectiveness of topical analgesics with and without Entonox for esophagogastroduodenoscopy: A randomized controlled trial

**DOI:** 10.1002/deo2.70107

**Published:** 2025-05-19

**Authors:** Papiroon Noitasaeng, Uayporn Kaosombatwattana, Rojsirin Chaiwong, Phongthara Vichitvejpaisal

**Affiliations:** ^1^ Department of Anesthesiology Faculty of Medicine Siriraj Hospital, Mahidol University Bangkok Thailand; ^2^ Division of Gastroenterology Department of Medicine, Faculty of Medicine Siriraj Hospital, Mahidol University Bangkok Thailand; ^3^ Department of Perioperative Nursing Faculty of Medicine Siriraj Hospital, Mahidol University Bangkok Thailand

**Keywords:** anesthesia, esophagogastroduodenoscopy, Inhalation Drug Administration, local anesthesia, patient satisfaction

## Abstract

**Objectives:**

Esophagogastroduodenoscopy (EGD) is vital for diagnosing and treating upper gastrointestinal symptoms, but patient discomfort and anxiety can affect procedural outcomes. This study aimed to compare the effectiveness of topical analgesics with and without Entonox during EGD in terms of procedural success, patient tolerance, and satisfaction.

**Methods:**

A prospective, randomized, double‐blinded, controlled trial. Patients were assigned to receive either 10% xylocaine spray in the control group (Group C) or 10% xylocaine spray combined with Entonox (Group E). Procedural success and patient comfort were evaluated using the Bath Gastroscopy Toleration Score and patient comfort scores, with scores of 0 or 1 indicating success. Satisfaction was measured using the numeric rating scale, where scores of 7 or higher indicated high satisfaction.

**Results:**

A total of 211 patients underwent EGD successfully (Group C = 106, Group E = 105). Patients in Group E demonstrated a significantly higher proportion of success rate (76.2% vs. 35.9%, *p* < 0.001), better toleration score (82.9% vs. 75.5%, *p* = 0.004), and better patient comfort score (86.7% vs. 39.6%, *p* < 0.001) compared to Group C. Endoscopists and patients in Group E expressed higher satisfaction levels (9 vs. 8, *p* < 0.01 and 9 vs. 8, *p* < 0.01). The side effects of Entonox were minimal. Notably, Group E had a lower proportion of high blood pressure and tachycardia during the procedure (*p* < 0.001).

**Conclusions:**

Combining Entonox with topical analgesics significantly improves tolerance, satisfaction, and procedural success during EGD, offering a safe and effective option for managing patient discomfort and anxiety.

## INTRODUCTION

Esophagogastroduodenoscopy (EGD) is a crucial endoscopic procedure for diagnosing and managing upper gastrointestinal conditions.^1^ It offers direct visualization of the mucosa, enables tissue acquisition, and allows for therapeutic interventions.^2^ However, EGD is often associated with discomfort and anxiety, which can significantly affect patient compliance and overall experience.^3^, ^4^ Anxiety before the procedure is common, and perioperative pain can exacerbate the situation, leading to panic attacks and reduced tolerance.^5^, ^6^ Given these challenges, it is essential to adopt strategies that alleviate patient fears and discomfort. Anesthesiologists play a critical role in managing these concerns, ensuring that patients undergo the procedure with minimal distress.

Various pharmacological and non‐pharmacological approaches are currently used to manage the discomfort associated with EGD. Topical anesthesia with local anesthetics, while effective to some extent, often fails to completely alleviate fear and anxiety. To improve patient tolerance, a combination of topical anesthesia and sedatives is frequently employed.^7^ Conscious sedation, which allows patients to tolerate pain and discomfort while maintaining adequate cardiorespiratory function and responsiveness, is a common practice during EGD.^8^, ^9^ Different classes of medications, including opioids, non‐steroidal anti‐inflammatory drugs, and inhalational agents, are used to manage pain during the procedure. Opioids, despite their efficacy, are associated with adverse effects such as nausea, vomiting, and respiratory depression.^10^ Non‐steroidal anti‐inflammatory drugs, while effective for inflammation‐related pain, avoid central nervous system side effects but can still pose risks for certain patients.^11^, ^12^ Inhalational agents, particularly nitrous oxide, offer a rapid onset of action and quick clearance, making them an attractive option for managing procedural pain.^13^

Entonox, a 50:50 mixture of nitrous oxide and oxygen, is widely recognized for its analgesic and sedative properties. It is commonly used in obstetric and surgical procedures due to its rapid onset, effective pain relief, and calming effects, all without inducing loss of consciousness.^14^, ^15^, ^16^, ^17^, ^18^, ^19^, ^20^ Entonox is easily administered via a mouthpiece, allowing patients to self‐regulate their intake during the procedure. Its side effects are generally minimal when used appropriately, with the most commonly reported adverse events including mild headaches, tingling sensations in the fingers and face, slight dizziness, and occasional nausea. These effects typically resolve quickly, with recovery times averaging around 30 min, making Entonox particularly suitable for outpatient procedures such as EGD.^14^, ^15^, ^16^, ^21^, ^22^, ^23^ The use of Entonox during EGD has the potential to enhance anesthesia care, improve patient satisfaction, and streamline procedural efficiency.

Previous studies have demonstrated the effectiveness of Entonox in pediatric endoscopy, where both endoscopists and nurses reported high satisfaction with its use.^24^ Similarly, in procedures like sigmoidoscopy and colonoscopy, Entonox has shown significant benefits in patient cooperation and comfort.^25^, ^26^, ^27^ However, research focusing specifically on EGD is limited, prompting the need for further investigation into its efficacy in this context.

This study aimed to compare the effectiveness of topical analgesics with and without Entonox during EGD. The primary objective was to assess the success rate of completing the endoscopic procedure while ensuring patient comfort and facilitating esophageal instrumentation by the endoscopist. Secondary objectives included evaluating endoscopist and patient satisfaction and assessing any potential side effects associated with Entonox use during EGD.

## MATERIALS AND METHODS

This double‐blind, single‐center, randomized controlled trial received approval from the Siriraj Institutional Review Board (Si‐IRB) with the Certificate of Approval number Si 262/2020. Additionally, it was registered with the Thai Clinical Trials Registry under the identifier TCTR20200721001. Before participating in the study, all subjects provided written informed consent.

### Participants

Initially, 212 patients scheduled for elective EGD were invited to participate in this study. Inclusion criteria included adults aged 18–70 with an American Society of Anesthesiologists (ASA) physical status classification of I and II.^28^ Patients needed to be capable of completing questionnaires and providing informed consent. Exclusion criteria encompassed patients with a body mass index over 35 kg/m^2^, those requiring general anesthesia or total intravenous anesthesia for EGD, individuals at risk of aspiration, and those with a history of severe medical conditions or respiratory issues like chronic obstructive pulmonary disease. Patients with a history of head injuries, maxillofacial injuries, recent surgeries, allergies to study drugs, or pregnancy were also excluded.

Patients were removed from the study if they withdrew consent, exhibited bronchospasm, or showed signs of lidocaine overdose or toxicity.

After providing written informed consent, patients were randomly assigned to one of two groups using a computer‐generated stratified randomization method in a block of four fashions. The factors for stratification included gender, age, American Society of Anesthesiologists classification, and body mass index to ensure a balanced distribution across the groups. The randomization number was kept in a sealed envelope and was unsealed only after the patient had written the consent. However, one patient in Group E reported feeling irritated by the topical analgesic and refused to continue participating, resulting in 211 participants completing the study.

### Intervention

Following randomization, patients received five puffs of lidocaine spray (10 mg/puff) at 5‐min intervals, totaling approximately 150 mg. The spray was applied to the tonsils, anterior pillars, and the base of the tongue. In the endoscopy room, patients in the control group (Group C) received oxygen through a mouthpiece, while those in the experimental group (Group E) inhaled Entonox.

To maintain blinding, the oxygen and Entonox canisters were concealed, and endoscopists were not permitted to enter the room until Entonox administration was complete. Patients self‐administered Entonox using a mouthpiece with a one‐way valve, ensuring a secure seal while taking deep breaths for eight breaths. This design minimized or prevented gas leakage, thereby reducing the risk of occupational exposure.

All patients were positioned in the left lateral position, blindfolded, and underwent the EGD procedure performed by an experienced endoscopist with over 1000 prior procedures. In cases where patients could not tolerate the procedure, additional sedation drugs were administered at the anesthesiologist's discretion.

To preserve blinding, the personnel responsible for outcome assessments were distinct from those involved in sedation management. The anesthesiologist, who was not part of the outcome assessment team, determined the need for sedation based on predefined criteria if patients were unable to tolerate the procedure, and sedation records were concealed from assessors. Additionally, post‐procedural evaluations were conducted by independent assessors who were unaware of the patient's group allocation or whether additional sedation had been administered.

No adverse effects related to Entonox administration or significant occupational exposure risks were observed among the medical personnel involved in this study. The use of a well‐sealed, one‐way valve system and the short duration of gas exposure contributed to the safety of both patients and healthcare providers.

### Outcome assessment

After the procedure, the endoscopists assessed the effectiveness of the EGD technique using the Bath Gastroscopy Toleration Score (0–4).^29^ Patients evaluated the severity of pain and discomfort experienced during the procedure using the patient comfort score (PCS, 0–4).^30^ The success rate was defined as a score of 0 and 1 in both the Bath Gastroscopy Toleration Score and PCS and the protocol was well‐performed. Satisfaction levels of both the endoscopists and patients were measured using the numerical rating scale (0–10) ranging from 0 (poor satisfaction) to 10 (excellent satisfaction), where scores of 7 or higher indicated high satisfaction.

The PCS and numerical rating scale were assessed 30 min after the completion of the EGD procedure to ensure consistency in data collection. Entonox has a rapid onset and elimination due to its low blood‐gas solubility coefficient, with an elimination half‐life of approximately 3–5 min after cessation of inhalation. Given this pharmacokinetic profile, it is expected that the effects of Entonox had entirely dissipated by the time the PCS and numerical rating scale were measured. Thus, these assessments reflect the patient's perception of procedural discomfort rather than any residual analgesic or sedative effects from Entonox.

Adverse events were recorded from the initiation of anesthesia through the completion of the procedure, with continuous monitoring of electrocardiography, heart rate (HR), pulse oximetry (SpO₂), and non‐invasive blood pressure (NIBP) at 5‐min intervals. Significant deviations in vital signs included BP or HR fluctuations >20% from baseline or pulse oximetry₂ <95%. Total procedure time, and any medication‐ or procedure‐related complications were also monitored.

Sedation depth was assessed using the Pasero Opioid‐Induced Sedation Scale^31^ during the procedure to evaluate patient responsiveness. Post‐procedure recovery was monitored in the recovery room for approximately one hour, with discharge readiness determined by the Modified Aldrete Score.^32^

### Statistical analysis

Categorical and continuous data were presented as frequency and percentage and mean ± standard deviation or median (interquartile range), respectively. The Chi‐square test and Fisher's exact tests were used to analyze categorical data while independent t‐tests and Mann‐Whitney U tests were used to compare continuous variables between the groups according to the distribution of data. The correlation between endoscopist and patient satisfaction was analyzed using Spearman's rho, with a *p*‐value of less than 0.05, which is considered statistically significant.

### Sample size calculation

The study aimed to compare the efficacy of local anesthesia alone versus the combination of local anesthesia and Entonox during EGD. The expected success rates were derived from previous studies investigating patient tolerance and comfort during unsedated gastroscopy. Specifically, the control group (local anesthesia alone) was anticipated to have a success rate of approximately 90%,^33^ while the experimental group (local anesthesia with Entonox) was expected to achieve a success rate approaching 99.9%. These estimates are based on findings from Qureshi et al., which demonstrated that approximately 92% of patients completed colonoscopies using Entonox without the need for additional sedation.^34^

With a significant level of 0.05 and a power of 80%, a sample size calculation determined that each group required 95 participants, leading to a total of 190 cases. To account for potential incomplete data or dropouts, 212 participants were enrolled.

## RESULTS

### Patient demographics

**FIGURE 1:**
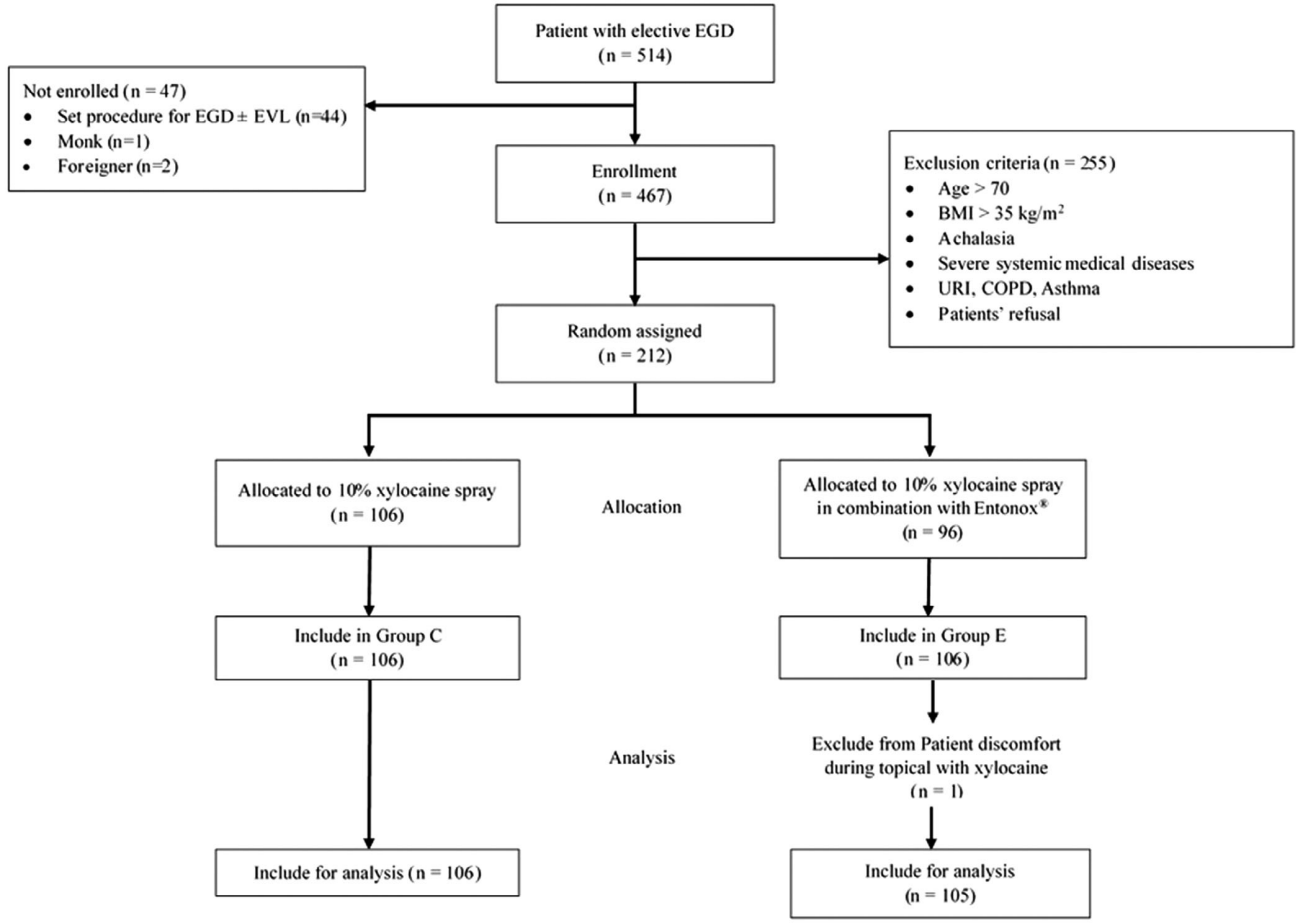
Flowchart depicting participant enrollment, allocation, and analysis in the study.

A total of 211 patients completed the study (Figure 1), with a mean age of 53.5 years, and 61.3% were female. Demographic data, including age, gender, American Society of Anesthesiologists physical status, body mass index, allergic reactions, underlying diseases, and EGD indications, were well‐matched between the groups (Table 1). Notably, there was a higher rate of patients with prior EGD experience in the group E (34.9% vs. 53.3%, *p* = 0.007).

**TABLE 1:** Patient demographic data.

Parameters	Control (*n* = 106) *n* (%)	Entonox (*n* = 105) *n* (%)	*p*‐value
Female	61 (57.5)	56 (53.3)	0.54
Age (years), median (range)	53 (38–61)	54 (44–62)	0.14
ASA			
I	33 (31.1)	27 (25.7)	0.38
II	73 (68.9)	78 (74.3)	
BMI (kg/m^2^), mean ± SD	23 ± 4	23 ± 4	0.99
Allergic conditions	22 (20.8)	17 (16.2)	0.39
Underlying diseases	73 (68.9)	78 (74.3)	0.38
*Hypertension*	32 (30.2)	30 (28.6)	0.80
*Diabetes mellitus*	14 (13.2)	16 (15.2)	0.67
Indications for EGD			
*Dyspepsia*	47 (44.3)	38 (36.2)	
*GERD*	18 (17.0)	13 (12.3)	
*Weight loss*	2 (1.9)	1 (1.0)	
*EV surveillance*	38 (35.8)	52 (49.5)	
*Anemia*	1 (1.0)	1 (1.0)	
History of previous EGD	37 (34.9)	56 (53.3)	0.007
Duration of procedure (min) (mean ± SD)	7.98 ± 4.3	6.35 ± 2.6	0.001*

Abbreviations: ASA, American Society of Anesthesiologists physical status classification; BMI, body mass index; EGD, esophagogastroduodenoscopy; EV, esophageal varices; GERD, gastroesophageal reflux disease.

*p‐value <0.05 was considered statistically significant.

### Success rate

All patients completed the EGD procedures, with group C taking slightly longer procedure time (7.98 ± 4.3 vs. 6.35 ± 2.6 min, *p* = 0.001). The success rate was higher in Group E compared to Group C (76.2% vs. 35.9%, *p* < 0.001; Table 2). Endoscopists reported greater tolerance (Bath Gastroscopy Toleration Score 0–1) in Group E (82.9% vs. 75.5%, *p* = 0.004), and patients also exhibited significantly higher comfort (PCS 0–1) in Group E (86.7% vs. 39.6%, *p* < 0.001). While additional sedation was required in both groups, it was more frequent in Group C (23.6% vs. 13.3%), though this difference was not statistically significant.

**TABLE 2:** The effectiveness score among endoscopists and patients.

Effectiveness score	Control (*n* = 106)	Entonox (*n* = 105)	*p*‐value
GTS			0.004*
0	27 (25.5%)	51 (48.6%)	
1	53 (50.0%)	36 (34.3%)	
2	18 (17.0%)	15 (14.3%)	
3	8 (7.5%)	3 (2.9%)	
4	–	–	
PCS			<0.001*
0	11 (10.4%)	40 (38.1%)	
1	31 (29.2%)	51 (48.6%)	
2	42 (39.6%)	11 (10.5%)	
3	12 (11.3%)	3 (2.9%)	
4	10 (9.4%)	0 (0%)	
Success criteria			
(GTS ≤ 1 and PCS ≤ 1)	38 (35.9%)	80 (76.2%)	<0.001*

*p‐value <0.05 was considered statistically significant.

Abbreviations: GTS, Gastroscopy Toleration Score; PCS, Patient Comfort Scores.

### Subgroup analysis based on prior EGD experience

Subgroup analysis was performed according to prior EGD experience. It was demonstrated that in patients with prior EGD experience, the success rate was significantly higher in Group E compared to Group C (83.9% vs. 29.7%, *p* = 0.003), with a relative risk (RR) of 3.9 in favor of Group E. Similarly, in patients without prior EGD experience, Group E also achieved a significantly higher success rate than Group C (67.3% vs. 39.1%, *p* < 0.001; Table 3). These findings suggest that Entonox remains effective regardless of prior EGD experience, with a notable benefit observed in both subgroups.

**TABLE 3:** The success rate of the procedure according to esophagogastroduodenoscopy experience.

Success rate	Control	Entonox	*p*‐value	Relative risk	*p*‐value (Tests of conditional independence for EGD experience)
All	(*n* = 106)	(*n* = 105)	<0.001	2.271	<0.001*
	38 (35.8%)	80 (76.2%)		(3.032, 1.700)	
Previous	(*n* = 37)	(*n* = 56)	0.003	3.917	
EGD experience	11 (29.7%)	47 (83.9%)		(6.902, 2.223)	
Without prior	(*n* = 69)	(*n* = 49)	<0.001	1.609	
EGD experience	27 (39.1%)	33 (67.3%)		(2.220, 1.167)	

*p‐value <0.05 was considered statistically significant.

### Satisfaction

**FIGURE 2:**
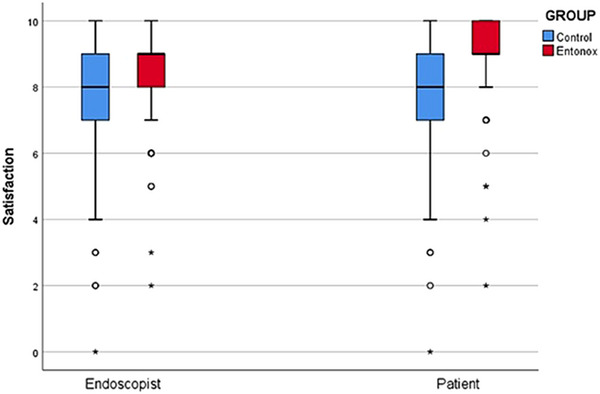
Correlation between endoscopist satisfaction and patient satisfaction scores.

Both endoscopists and patients in Group E expressed higher satisfaction with the procedure and drug administration (*p* < 0.01). Patients in Group E showed a strong preference for using Entonox in future procedures (*p* < 0.01; Table 4). The Spearman's rho correlation^35^ between endoscopist and patient satisfaction was significant (0.424 in Group C vs. 0.448 in Group E, *p* < 0.001), indicating a strong relationship between the two (Figure 2).

**TABLE 4:** The satisfaction score and the choice of anesthesia for the next procedure.

Satisfaction score	Control (*n* = 106)	Entonox (*n* = 105)	*p*‐value
Endoscopists	8 (7–9)	9 (8,9)	<0.01*
Patients	8 (7–9)	9 (9,10)	<0.01*
Treatment of choice, n (%)			<0.01*
Yes	83 (78.3%)	100 (95.2%)	
No	22 (20.8%)	5 (4.8%)	
Uncertainty	1 (0.9%)	0 (0%)	

*p‐value <0.05 was considered statistically significant.

### Efficacy

Efficacy analysis revealed that elevated blood pressure and tachycardia were less prevalent in Group E. Patients in Group E experienced significantly lower rates of hypertension (63% vs. 18%) and tachycardia (36% vs. 11%). These differences were statistically significant (*p* < 0.001; Table 5).

**TABLE 5:** The adverse events of medication and procedure.

Complications	Control (*n* = 106)	Entonox (*n* = 105)	*p*‐value
High blood pressure	67 (63%)	19 (18%)	<0.001*
Bradycardia	1 (1%)	0 (0%)	1.00
Tachycardia	38 (36%)	12 (11%)	<0.001*
Dizziness	2 (2%)	0 (0%)	0.50

*p‐value <0.05 was considered statistically significant.

### Safety

No serious complications, including hypotension, cardiac arrhythmia, upper airway obstruction, hypoxia, or vomiting, were reported in either group. In Group E, minor adverse events such as transient nausea (4.8%) and dizziness (3.3%) were observed, while dizziness was reported by two patients in Group C (2%; Table 5). All symptoms were self‐limiting and resolved without intervention.

## DISCUSSION

This study robustly supports the use of Entonox alongside topical anesthesia during EGD procedures, confirming its significant benefits in enhancing patient comfort and cooperation. The addition of Entonox to the standard topical anesthesia with lidocaine has shown a significantly higher tolerance and satisfaction than those treated with topical anesthesia alone without accompanying an increase in adverse effects. This outcome underlines the positive impact of Entonox on the overall patient experience, regardless of prior EGD experience, supporting its adoption as a standard adjunct to topical anesthesia in such procedures.

While topical anesthesia with lidocaine has been acknowledged for its efficacy, it often falls short of fully addressing patient anxiety and apprehension associated with EGD.^36^, ^37^, ^38^, ^39^ This, in turn, could potentially affect the endoscopist's ability to complete the procedure successfully. In response to this concern, combining Entonox with topical analgesics in urological day case procedures (prostate biopsy, flexible cystoscopy, and extracorporeal shock wave lithotripsy) can enhance patient comfort further, facilitating a more relaxed experience and improving cooperative behavior during procedures.^18^

Historically, the integration of Entonox into EGD was limited, possibly due to concerns over the procedure's duration and the need for more profound sedation. Nevertheless, recent advancements in pharmacology and more excellent proficiency among endoscopists and anesthesiologists have allowed for its broader application in these settings. Our results complied with previous studies using Entonox for gastrointestinal endoscopy.^24^ A randomized study focusing on flexible sigmoidoscopy showed that stress response, as demonstrated by the HR variability metric, was reduced in patients who inhaled Entonox during the procedure.^25^ This finding confirmed the result of our study that the prevalence of tachycardia was lower in the Entonox group. Also, Entonox poses benefits in terms of a reduction in hypertension and tachycardia when compared with topical analgesia.

For more extensive procedures like colonoscopies that require prolonged sedation, it may be feasible to administer repeated doses or continuous use of Entonox.^40^ Moreover, Entonox provided similar pain control and patient/endoscopist satisfaction compared with propofol or midazolam‐fentanyl sedation in elective colonoscopy.^22^, ^26^, ^41^ This strategy can effectively bridge the gap between the necessity for continuous deep sedation and the desire to maintain patient comfort. The British Society of Gastroenterology guidelines on sedation in gastrointestinal endoscopy also recommended using inhalational agents as an alternative to sedation for endoscopy.^42^ Entonox was safe and effective for colonoscopy, as we demonstrated earlier. However, with the concern of staff exposure and greenhouse gas emissions of nitrous oxide, a protective measure is required to generalize Entonox use in clinical practice.

Regarding safety, severe complications were uncommon in both study groups, with minimal reports of dizziness and no significant disparities in adverse events between them. Interestingly, the Entonox group exhibited lower hypertension and tachycardia, likely attributable to its mild analgesic and sedative properties. This underscores the safety and efficacy of Entonox in reducing physiological stress responses during EGD procedures. Additional research, such as the studies by Galeotti et al. and Abril‐Sánchez et al., supports that inhalation‐conscious sedation with Entonox effectively reduces HR fluctuations and stress during medical procedures.^9^, ^43^ Overall, our findings underscore the safety and efficacy of Entonox over topical analgesia regarding mitigation of physiological stress during EGD and postprocedural complications.

The study's limitations include the higher proportion of patients with prior EGD experience in the Entonox group, which may have influenced the results. Additionally, the absence of long‐term follow‐up limits the ability to assess the sustained effects of Entonox on patient outcomes. Future research should address these limitations by incorporating stratified randomization to balance prior EGD experience and including long‐term follow‐up to evaluate the durability of the observed benefits.

## CONCLUSION

Integrating Entonox with topical anesthesia during EGD significantly improves patient tolerance and satisfaction, making it a valuable adjunct in managing EGD discomfort. The study findings support the broader adoption of Entonox in clinical practice, particularly in settings where patient comfort and quick recovery are priorities. Further research is needed to explore the long‐term benefits and potential applications of Entonox in other endoscopic and non‐endoscopic procedures.

## CONFLICT OF INTEREST STATEMENT

None.

## ETHICS STATEMENT

**Approval of the research protocol by an Institutional Reviewer Board**: This prospective study and randomized controlled trial received approval from the Siriraj Institutional Review Board (Si‐IRB) with the Certificate of Approval number (COA number) Si 262/2020.

## PATIENT CONSENT STATEMENT

All patients provided written informed consent prior to participating in the study.

## CLINICAL TRIAL REGISTRATION

This study was registered with the Thai Clinical Trials Registry under the identifier TCTR20200721001.
